# Coronary no-reflow and adverse events in patients with acute myocardial infarction after percutaneous coronary intervention with current drug-eluting stents and third-generation P2Y_12_ inhibitors

**DOI:** 10.1007/s00392-023-02340-y

**Published:** 2023-11-14

**Authors:** Gjin Ndrepepa, Salvatore Cassese, Erion Xhepa, Michael Joner, Hendrik B. Sager, Sebastian Kufner, Karl-Ludwig Laugwitz, Heribert Schunkert, Adnan Kastrati

**Affiliations:** 1 Department of Cardiology, Deutsches Herzzentrum München, Technische Universität München, Lazarettstrasse 36, 80636 Munich, Germany; 2 German Center for Cardiovascular Research (DZHK), Partner Site Munich Heart Alliance, Munich, Germany; 3 Medizinische Klinik und Poliklinik Innere Medizin I (Kardiologie, Angiologie, Pneumologie), Klinikum rechts der Isar, Technische Universität München, Munich, Germany

**Keywords:** Acute myocardial infarction, Coronary no-reflow, Percutaneous coronary intervention, Prognosis

## Abstract

**Background:**

The frequency and prognostic value of coronary no-reflow (CNR) was investigated in studies that have used an outdated reperfusion therapy in terms of stent technology and antithrombotic drugs. We assessed the association of CNR with adverse outcomes in patients with acute myocardial infarction (AMI) undergoing percutaneous coronary intervention (PCI) with drug-eluting stents (DES) and newer antithrombotic drugs, ticagrelor or prasugrel.

**Methods:**

This study included 3100 patients with AMI who underwent PCI with current DES and third-generation P2Y_12_ inhibitors. CNR was defined as Thrombolysis in Myocardial Infarction (TIMI) blood flow grade ≤ 2 at the end of PCI. The primary end point was 1-year incidence of net adverse clinical and cerebral events—a composite end point of death of any cause, myocardial infarction, stroke or major bleeding.

**Results:**

CNR was diagnosed in 130 patients (4.2%). The primary end point occurred in 28 patients in the CNR group and 354 patients in the reflow group (cumulative incidence 23.2% and 12.8%; adjusted hazard ratio = 1.53, 95% confidence interval 1.01–2.33; *P* = 0.049). The 1-year incidences of death or myocardial infarction (14.6% vs. 7.6%; *P* = 0.003), myocardial infarction (8.8% vs. 3.9%; *P* = 0.007) and major bleeding (10.9% vs. 5.6%; *P* = 0.008) were significantly higher in patients with CNR than patients with reflow. The risk of adverse events in patients with CNR was highest within the first 30 days after PCI.

**Conclusion:**

In patients with AMI undergoing PCI with current DES and third generation P2Y_12_ receptor inhibitors, CNR was associated with a higher risk of adverse outcomes at 1 year.

**Graphical abstract:**

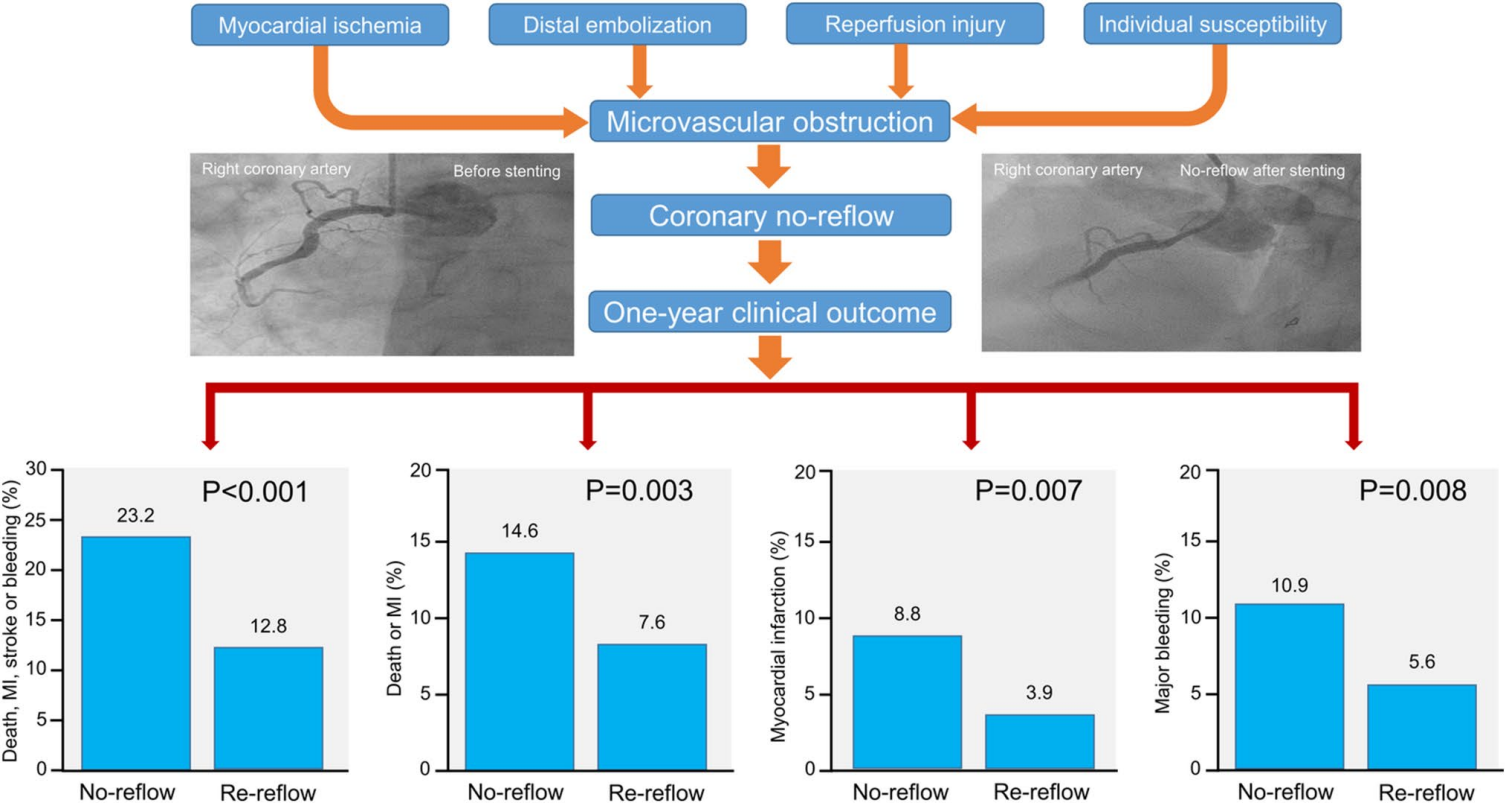

**Supplementary Information:**

The online version contains supplementary material available at 10.1007/s00392-023-02340-y.

## Introduction

Coronary no-reflow (CNR) is defined as inadequate myocardial reperfusion following successful opening of an occluded coronary artery without residual flow-impeding mechanical obstacle in the infarct-related artery [1]. The main pathophysiological mechanism of CNR is microvascular obstruction (MVO) that develops in patients with acute myocardial infarction (AMI) as a consequence of myocardial ischemia, spontaneous or iatrogenic distal embolization and reperfusion-related injury that may be more common in patients with pre-existing microvascular dysfunction [2, 3]. CNR was reported to occur in between 5% [4] and 32% [5] of patients after primary percutaneous coronary intervention (PCI) by Thrombolysis in Myocardial Infarction (TIMI) criteria. CNR markedly offsets the benefits of reperfusion in patients with AMI due to its association with adverse left ventricular remodeling, new or worsening of congestive heart failure and reduced survival [6–8]. The prognostic value of CNR has mostly been investigated in studies that have used an outdated reperfusion therapy in terms of stent technology and antithrombotic drugs. CNR has mostly been studied in terms of its association with adverse outcomes, such as left ventricular adverse remodeling, congestive heart failure or mortality and the association between CNR and other adverse outcomes, such as myocardial infarction, bleeding, stroke or stent thrombosis after PCI has not been investigated. In addition, the prognostic impact of CNR has mostly been studied in patients with ST-segment elevation myocardial infarction (STEMI) and the evidence on the association of CNR with adverse outcomes in patients with non-ST-segment elevation myocardial infarction (NSTEMI) is limited [9]. Finally, whether the strength of association between CNR and outcome differs over time after PCI is unknown. Against this background, we undertook this study to assess the association of CNR with adverse outcomes in patients with AMI undergoing PCI with current generations of drug-eluting stents (DES) and newer antithrombotic drugs, ticagrelor or prasugrel.

## Methods

### Patients

This study included 3100 patients with STEMI (n = 1568 patients) or NSTEMI (*n* = 1532 patients) who underwent PCI in the setting of the Intracoronary Stenting and AntiThrombotic Regimen: Rapid Early Action for Coronary Treatment 5 trial (ISAR-REACT 5; Clinical Trial Registration: NCT01944800) between September 2013 and February 2018 [10]. STEMI was diagnosed if the patient presented (or had) with chest discomfort suggestive of myocardial ischemia lasting more than 20 min within 24 h prior to randomization associated with ST-segment elevation ≥ 1 mm in ≥ 2 extremity leads or ≥ 2 mm in ≥ 2 contiguous precordial leads or left bundle branch block of new onset in the electrocardiogram. NSTEMI was diagnosed in patients with symptoms consistent with an acute coronary syndrome (chest pain and/or electrocardiographic changes [transient ST elevation, ST depression, or new T wave inversions] not consistent with STEMI) and elevation of cardiac troponin. Patients were randomly assigned to receive ticagrelor or prasugrel in the setting of the primary trial. Ticagrelor was started at a loading dose of 180 mg and continued at a maintenance dose of 90 mg twice daily. Prasugrel was started at a loading dose of 60 mg and continued at a maintenance dose of 10 mg once per day. In patients > 75 years of age or those with a body weight of < 60 kg, a reduced dose of prasugrel (5 mg once daily) was recommended [11]. In both study arms, aspirin was started at a loading dose of 150–300 mg (intravenously or orally as a chewed drug) and continued at a maintenance dose of 75–100 mg once daily. The study conforms to the declaration of Helsinki. The study protocol was approved by the local ethics committee at each participating center.

### Study definitions

Baseline (before the intervention) and postprocedural (after the intervention) TIMI flow grade was defined according to the TIMI Group definitions [12]. Angiographic CNR was defined as a postprocedural TIMI flow grade of ≤ 2 with no angiographic evidence of flow-limiting residual stenosis (< 50%), coronary vessel dissection, spasm or apparent thrombus in the treated coronary artery in the coronary angiography performed at the end of PCI procedure. Only persistent CNR at the end of PCI but not transient blood flow fluctuations during the PCI procedure was considered.

Cardiovascular risk factors—arterial hypertension, diabetes mellitus, hypercholesterolemia and smoking—were defined as per guideline-recommended criteria at the time of patient’s recruitment in the primary trial. Body mass index was calculated using patient’s height and weight measured during the hospital course. Global left ventricular ejection fraction was measured using the area-length method on left ventricular angiograms according to the Sandler and Dodge method [13]. Cardiac troponin T (hsTnT) was measured using a high-sensitivity assay (Roche Diagnostics, Basel, Switzerland) on a cobas e 411 immunoanalyzer (Roche Diagnostics). The 99th upper reference limit (URL) is 14 ng/L. Creatine kinase myocardial band (CK-MB) in plasma was determined using a COBAS INTEGRA system (Roche Diagnostics). Serum creatinine was measured using a kinetic colorimetric assay according to the compensated Jaffe method.

### Outcomes and follow-up

The primary end point was the 1-year incidence of net adverse clinical and cerebral events (NACCE)—a composite end point of death of any cause, myocardial infarction, stroke or major bleeding. The primary end point, a composite of death, myocardial infarction or stroke, a composite of death or myocardial infarction, cardiovascular death, individual components of the primary end point and definite or probable stent thrombosis at 30 days and 1 year were also assessed. Cardiovascular death and definite or probable stent thrombosis were defined according to the Academic Research Consortium criteria [14]. Myocardial infarction was diagnosed according to the 3rd Universal Definition of Myocardial Infarction criteria [15]. Major bleeding was defined as type 3–5 bleeding according to the Bleeding Academic Research Consortium (BARC) criteria [16]. Stroke was defined as the new onset of focal or global neurological deficit caused by ischemia or hemorrhage within or around the brain lasting for more than 24 h or leading to death. The diagnosis of stroke was confirmed by brain imaging tests or autopsy. All outcomes were analyzed in the intention-to-treat population. All adverse events analyzed in this study were adjudicated by event adjudication committee in the setting of primary trial.

The clinical follow-up was scheduled at one month (± 10 days), 6 months (± 1 month) and 1 year (± 1 month) in the setting of primary trial [10]. Patients were monitored either via hospital, outpatient visits or through telephone and structured follow-up letters. All adverse events were adjudicated by members of the event adjudication committee, who were unaware of clinical or angiographic data of the patients.

### Statistical analysis

Continuous variables are presented as mean ± standard deviation or median [25th–75th percentiles] and compared using the *t*-test or Wilcoxon rank sum test, when appropriate. The distribution pattern of continuous data was assessed using the Kolmogorov–Smirnov test. Categorical variables are shown as counts and proportions (%) and compared using the chi-squared test. Correlates of CNR are assessed by multivariable logistic regression model. All baseline characteristics shown in Table 1 and Supplemental Table S1 are included in the model. The cumulative incidences of the primary end point and death were assessed by computing the Kaplan–Meier estimates of event-free survival. Differences between the groups were compared using the univariate Cox proportional hazards model. For all end points except for death or end points that incorporated death, the cumulative incidence functions were computed after accounting for the competing risk of death. The CNR-by-clinical presentation (STEMI or NSTEMI) and CNR-by-drug (ticagrelor or prasugrel) interactions were assessed. The multivariable Cox proportional hazards model was used to assess the correlates of the primary end point. Covariates entered into the multivariable Cox proportional hazards model were selected using the least absolute shrinkage and selection operator (LASSO) regression method (R-package “glmnet”, version 2.0-13). The following variables were entered into the model: CNR, age, sex, diabetes mellitus, hypercholesterolemia, systolic blood pressure, heart rate, baseline creatinine, history of myocardial infarction, multivessel disease, vascular access (radial vs. femoral artery), target vessel, angiographic left ventricular ejection fraction, complex lesions, stenting (versus angioplasty alone) and randomization to drug loading time interval. Missing values of baseline data were imputed by predictive mean matching. The C statistic of the multivariable Cox proportional hazards models for the primary end point without CNR (with baseline variables) and with CNR (baseline variables plus CNR) were calculated and compared using the CompareC package. The statistical analysis was performed using the R 4.1.0 Statistical Software (The R foundation for Statistical Computing, Vienna, Austria). A two-sided *P* < 0.05 was considered to indicate statistical significance.

**Table 1:** Baseline characteristics

Characteristic	No-reflow (*n* = 130)	Reflow (*n* = 2970)	*P* value
Age (years)	66.6 ± 11.8	64.1 ± 12.1	0.020
Age ≥ 75 years	41 (31.5)	678 (22.8)	0.028
Women	29 (22.3)	624 (21.0)	0.806
Diabetes mellitus	28 (21.5)	640 (21.6)	1.00
On insulin therapy	10 (7.7)	202 (6.8)	0.830
Current smoker	33/129 (25.6)	1094/2958 (37.0)	0.008
Arterial hypertension	73 (56.2)	2010/2963 (67.8)	0.005
Hypercholesterolemia	70 (53.8)	1656 (55.9)	0.667
Body mass index (kg/m^2^)	27.6 ± 4.10	27.8 ± 4.50	0.504
History of myocardial infarction	26 (20.0)	410/2968 (13.8)	0.047
History of CABG	22 (16.9)	144/2968 (4.8)	< 0.001
History of PCI	32 (24.6)	564/2967 (19.0)	0.112
Type of myocardial infarction			0.001
STEMI (%)	84 (64.6)	1484 (50.0)	
NSTEMI (%)	46 (35.4)	1486 (50.0)	
Systolic blood pressure (mmHg)	142.0 ± 28.2	142.0 ± 25.0	0.943
Diastolic blood pressure (mmHg)	84.0 ± 16.0	82.2 ± 14.3	0.225
Heart rate (beats/min)	79.0 ± 19.3	76.8 ± 15.6	0.213
Serum creatinine (µmol/l)	90.1 ± 31.8	87.5 ± 28.7	0.365
Peak creatine kinase MB (U/L)	108.0 [43.5;236.0]	68.0 [28.0;171.0]	< 0.001
Antithrombotic drugs on admission			
Aspirin	889 (29.9)	43 (33.1)	0.504
Clopidogrel	105 (3.5)	5 (3.8)	0.807
Symptom onset to randomization time (hours)	8.00 [3.17;20.0]	7.33 [2.58;19.50]	0.538
Randomization to drug loading time (min)	5.0 [1.0;20.0]	9.0 [1.0;39.0]	0.011
Study drug			0.075
Prasugrel	55 (42.3)	1505 (50.7)	
Ticagrelor	75 (57.7)	1465 (49.3)	

Data are mean ± standard deviation, median [25th–75th percentiles] and counts (%)

*CABG* coronary artery bypass graft, *MB* myocardial band, *PCI* percutaneous coronary intervention, *NSTEMI* non-ST-segment elevation myocardial infarction, *STEMI* ST-segment elevation myocardial infarction

## Results

### Baseline data

Overall, angiographic CNR at the end of PCI procedure occurred in 130 patients (4.2%). CNR occurred in 84 patients with STEMI and 46 patients with NSTEMI (5.4% vs. 3.0%; odds ratio [OR] = 1.83, 95% confidence interval 1.27–2.64; *P* = 0.001). CNR occurred in 55 patients assigned to prasugrel and 75 patients assigned to ticagrelor (3.5% vs. 4.9%; OR = 0.71 [0.50–1.02]; *P* = 0.062). Baseline data are shown in Table 1. Patients with CNR were older (including patients ≥ 75 years of age) and less likely to be current smokers or have arterial hypertension compared with patients with reflow. Patients with CNR were more likely to have had previous myocardial infarction or coronary artery bypass surgery, had a higher proportion of patients with STEMI and had shorter randomization to drug loading time interval compared with patients with reflow. Other baseline data appear to differ little between patients with CNR or reflow. Procedural data are shown in Supplementary Table S1. Patients with CNR were more likely to have had femoral artery used for vascular access, had lower left ventricular ejection fraction, had more frequent complex lesions and higher proportions of patients with baseline TIMI flow grade of 0 and 1 than patients of the reflow group. In addition, patients with CNR were less likely to have received drug-eluting stents, had less often more than one lesion treated and had higher proportions of patients loaded with aspirin and of those receiving periprocedural glycoprotein 2b/3a inhibitors than patients of the reflow group. There were also differences between the groups with respect to the treated vessel. At discharge, patients with CNR were less likely to be prescribed aspirin and more likely to be prescribed clopidogrel and oral anticoagulants than patients of the reflow group (Supplementary Table S2).

### Correlates of CNR

Correlates of CNR were assessed using the multivariable logistic regression model. The model identified no history of arterial hypertension (*P* < 0.001), elevated heart rate (*P* = 0.032), history of coronary artery bypass surgery (*P* < 0.001), lower baseline TIMI flow grade (*P* < 0.001) and plain balloon angioplasty (*P* < 0.001) as independent correlates of CNR. Older age was close to reaching the level of statistical significance (*P* = 0.070).

### Clinical outcome

Clinical outcomes at 30 days and 1 year are shown in Table 2. At 30 days, NACCE point occurred in 26 patients in the CNR group and 214 patients in the reflow group (cumulative incidence, 21.3% and 7.4%, respectively; hazard ratio [HR] = 3.16, 95% confidence interval [CI] 2.10–4.74; *P* < 0.001). The 30-day incidences of death, myocardial infarction or stroke, death or myocardial infarction, death, cardiovascular death, myocardial infarction and major bleeding were significantly higher in patients with CNR compared with patients with reflow. There were no significant differences with respect to the 30-day incidence of stroke or stent thrombosis in patients with CNR versus those with reflow (Table 2).

**Table 2:** Outcomes of patients with and without angiographic coronary no-reflow

Outcome	No-reflow (*n* = 130)	Reflow (*n* = 2970)	Hazard ratio [95% CI]	*P* value
30-day outcome				
NACCE	26 (21.3)	214 (7.4)	3.16 [2.10–4.74]	< 0.001
Death, myocardial infarction or stroke	15 (11.5)	110 (3.7)	3.26 [1.90–5.59]	< 0.001
Death or myocardial infarction	14 (10.8)	97 (3.3)	3.44 [1.97–6.03]	< 0.001
Death of any cause	9 (6.9)	56 (1.9)	3.78 [1.87–7.65]	< 0.001
Cardiovascular death	8 (6.2)	50 (1.7)	3.76 [1.78–7.93]	< 0.001
Myocardial infarction	7 (5.5)	46 (1.6)	3.60 [1.63–7.98]	0.002
Stroke	2 (1.6)	16 (0.5)	2.93 [0.67–12.76]	0.151
Major bleeding^a^	14 (10.9)	101(3.4)	3.34 [1.91–5.84]	< 0.001
Definite or probable ST	3 (2.3)	30 (1.0)	2.36 [0.72–7.75]	0.155
One-year outcome				
NACCE	28 (23.2)	354 (12.8)	2.12 [1.44–3.11]	< 0.001
Death, myocardial infarction or stroke	20 (15.4)	237 (8.1)	2.04 [1.29–3.22]	0.002
Death or myocardial infarction	19 (14.6)	223 (7.6)	2.05 [1.29–3.28]	0.003
Death of any cause	10 (7.7)	126 (4.3)	1.87 [0.99–3.56]	0.056
Cardiovascular death	8 (6.2)	94 (3.2)	2.00 [0.98–4.13]	0.059
Myocardial infarction	11 (8.8)	112 (3.9)	2.36 [1.27–4.38]	0.007
Stroke	2 (1.6)	26 (0.9)	1.81 [0.43–7.64]	0.417
Major bleeding	14 (10.9)	164 (5.6)	2.08 [1.21–3.59]	0.008
Definite or probable ST	3 (2.3)	39 (1.3)	1.82 [0.56–5.90]	0.317

Data are cumulative incidences of death and end points that include death and cumulative incidences after accounting for competing risk of death for other outcomes

*CI* confidence interval, *NACCE* net adverse clinical and cerebral events, *ST* stent thrombosis

^a^Bleeding Academic Research Consortium (BARC) class 3–5 bleeding

**Fig. 1:**
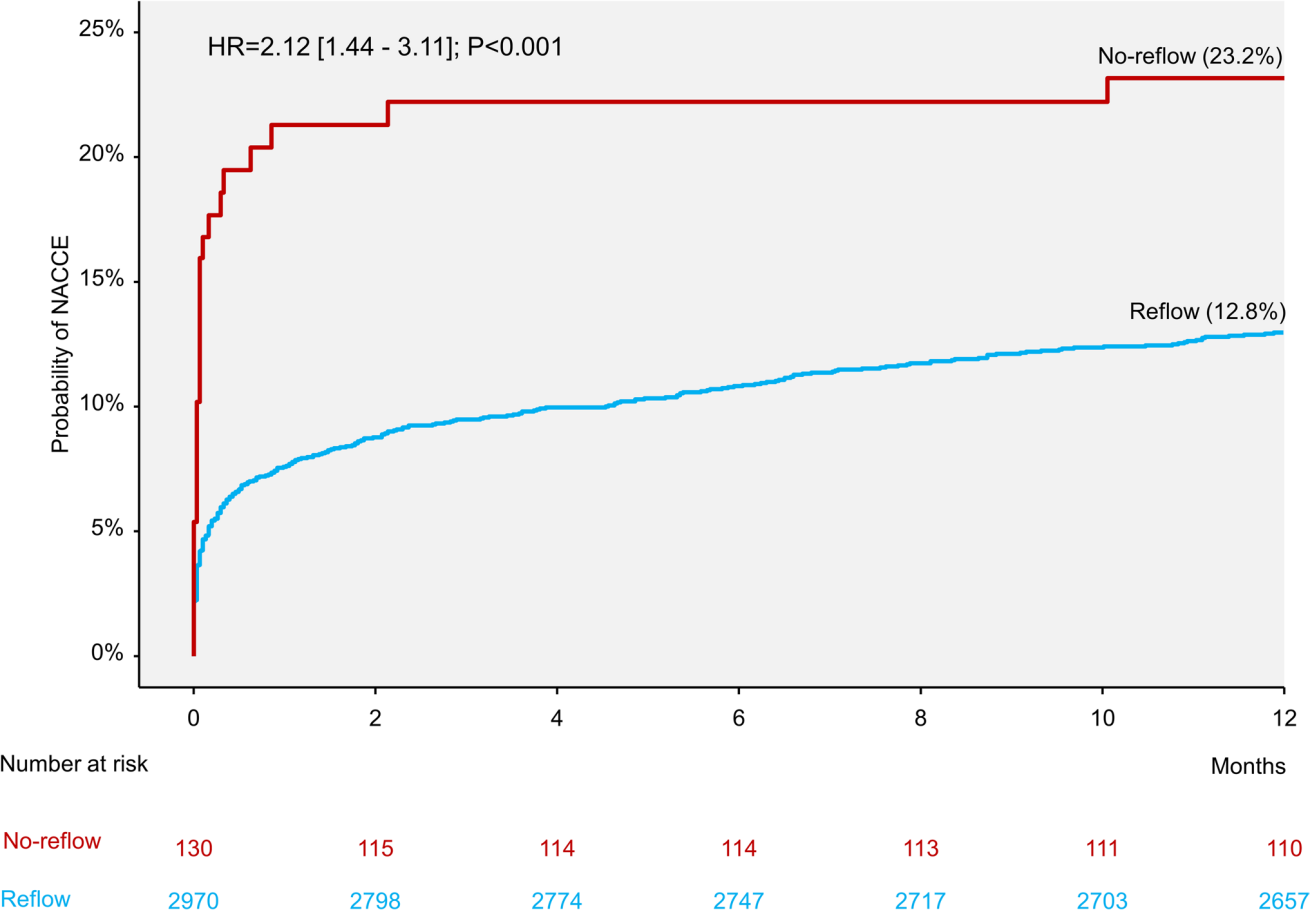
One-year incidence of net adverse clinical and cerebral events (NACCE) in patients with coronary no-reflow and reflow.* HR* hazard ratio

**Fig. 2:**
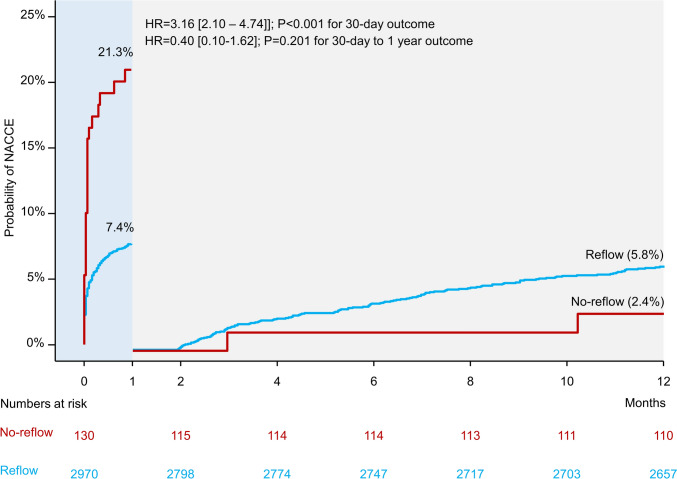
Landmark analysis showing probability of early (within 30 days) and late (30 days–1 year) net adverse clinical and cerebral events (NACCE) in patients with coronary no-reflow and reflow. * HR* hazard ratio

At 1 year, the primary end point (NACCE) occurred in 28 patients in the CNR group and 354 patients in the reflow group (cumulative incidence 23.2% and 12.8%, respectively; hazard ratio [HR] = 2.12 [1.44–3.11]; *P* < 0.001; Fig. 1). The 1-year incidences of death, myocardial infarction or stroke, death or myocardial infarction, myocardial infarction or major bleeding were significantly higher in patients with CNR compared with patients with reflow. Types of myocardial infarction are shown in Supplementary Table S3. The differences in the incidence of death or cardiovascular death were close to reaching the statistical significance. There were no significant differences with respect to the 1-year incidence of stroke or stent thrombosis in patients with CNR versus those with reflow (Table 2). The landmark analysis showed that the majority of events occurred within the first 30 days after the PCI procedure, whereas the differences between the groups were not significant in the 30-day to 1-year time interval (Fig. 2). Time-to-event curves of the incidence of death, myocardial infarction or stroke and landmark analysis for this outcome are shown in Supplementary Figures S1 and S2.

Overall, there was no significant CNR-by-clinical presentation (STEMI or NSTEMI) interaction (*P*_int_ = 0.074) with respect to the risk for NACCE. The CNR-by drug (ticagrelor or prasugrel) interaction was significant (*P*_int_ = 0.035) showing that CNR was associated with a higher risk of 1 year NACCE in patients treated with ticagrelor than patients treated with prasugrel. In a Cox proportional hazards model that included CNR, clinical presentation (STEMI and NSTEMI) and randomization to drug loading time interval, CNR was associated with the 1-year risk of NACCE with a HR = 2.10 [1.43–3.10] (STEMI: HR = 1.56 [0.92–2.64]; NSTEMI: HR = 3.20 [1.81–5.62]) without clinical presentation-by-randomization to drug loading time interval interaction (*P*_int_ = 0.371).

### Results of the multivariable analysis

The association between CNR and the risk of NACCE was adjusted in the multivariable Cox proportional hazards model (see methods for the variables that were entered into the model). The model showed that CNR was an independent correlate of NACCE at 30 days (adjusted HR = 1.85 [1.17–2.93]: *P* = 0.008) and 1 year (adjusted HR = 1.53 [1.01–2.33]; *P* = 0.049). The correlates of 30-day and 1-year NACCE are shown in the Supplementary Table S4. The C-statistic(s) of the multivariable models without and with inclusion of CNR were 0.736 [0.710–0.763] and 0.738 [0.712–0.765], *P* = 0.260) with respect to the discrimination for the primary end point.

## Discussion

In this study, we assessed the association between angiographic CNR and 30-day and 1-year adverse outcomes in patients with AMI undergoing PCI with current generation of DES and third generation P2Y_12_ receptor inhibitors. The main findings of the study can be summarized as follows: (1) in patients with AMI undergoing PCI with current generation of DES and third generation P2Y_12_ receptor inhibitors, ticagrelor or prasugrel, CNR was associated with a higher risk of adverse outcomes at 30 days and 1 year after PCI. Thus, advanced coronary stents and newer antiplatelet drugs cannot offset the association of CNR with adverse outcomes. (2) CNR was less frequent in patients with NSTEMI compared with patients with STEMI; however, there was no significant CNR-by-clinical presentation interaction in terms of adverse outcomes showing that CNR was associated with a higher risk of adverse outcomes in both types of AMI. 3) CNR was associated with a higher risk of thrombotic (death, myocardial infarction, stroke or stent thrombosis) and bleeding (BARC 3–5 bleeding) events at 30 days and 1 year after PCI. Although CNR was associated with a higher risk of stroke and stent thrombosis, the differences according to CNR did not reach the level of statistical significance due to small number of events. (4) The association between CNR and adverse events was strongest within the first 30 days and the risk for adverse events between 30 days and 1 year appears to differ little according to CNR.

**Fig. 3:**
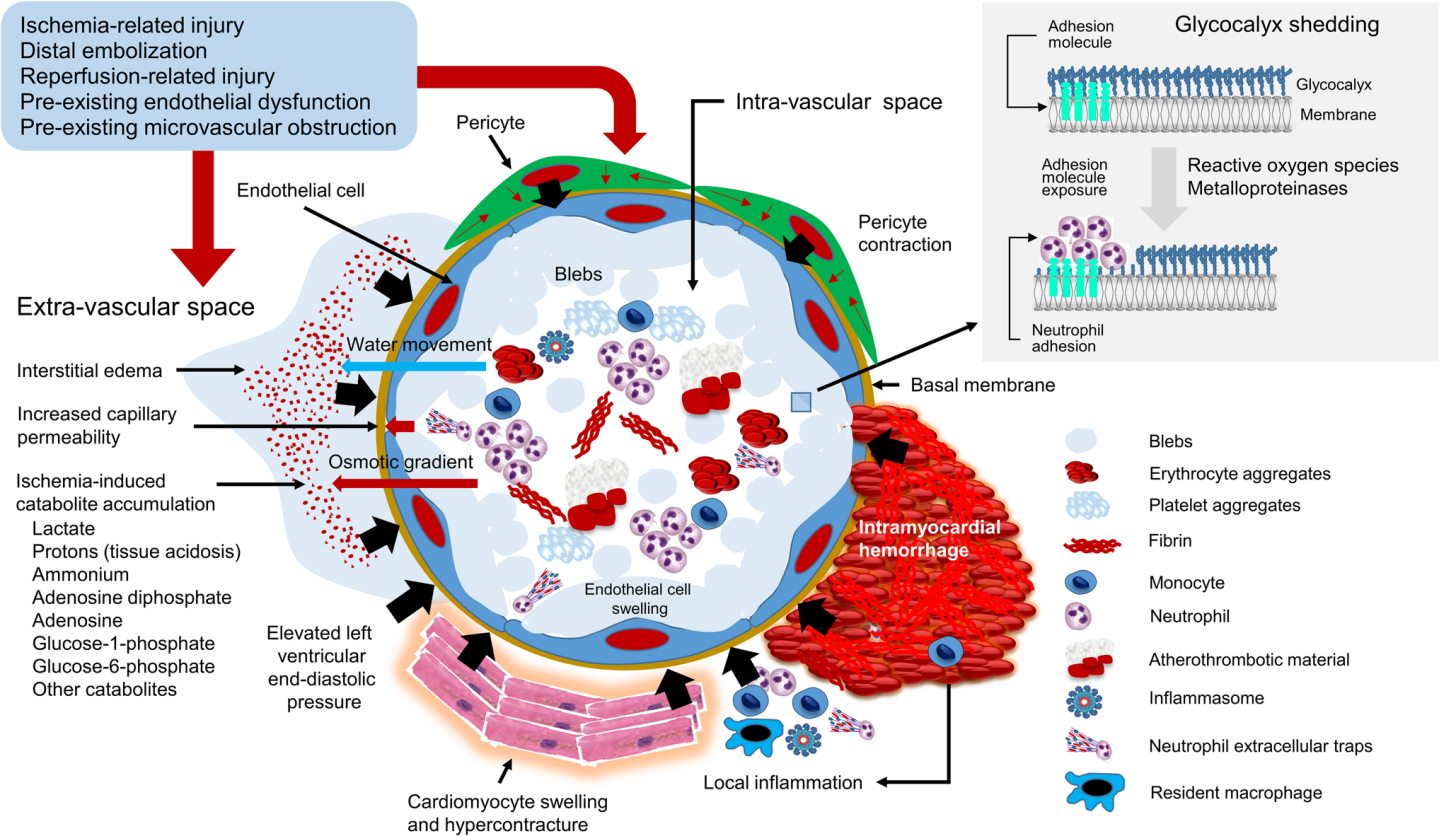
Pathophysiology of coronary no-reflow. The cross-sectional view at the microcirculation (capillary) level shows events that develop in the extravascular and intravascular space leading to microvascular obstruction and coronary no-reflow

CNR represents a manifestation of MVO [1, 2, 4] that results from intra-vascular and extra-vascular obstruction at the level of microcirculation induced by myocardial ischemia, distal embolization and reperfusion-related injury (Fig. 3). Multiple studies have confirmed an association between CNR and adverse outcomes in patients with AMI. The underlying mechanisms of the association between CNR and adverse outcomes include severe and prolonged myocardial ischemia associated with CNR [17], conditions associated with pre-existing endothelial dysfunction being more frequent in patients with CNR [3, 17], delayed removal of necrotic debris and blocked arrival of cytokines involved in tissue healing by CNR [18] and frequent association of CNR with intramyocardial hemorrhage [19, 20]. Ample evidence supports an association of CNR with adverse events, such as adverse remodelling of left ventricle, new or worsening congestive heart failure and increased risk of mortality [6–8]. Our study expands the list of adverse outcomes associated with CNR after reperfusion in patients with AMI. We found that patients with CNR had a higher risk of ischemic/thrombotic and bleeding complications after PCI. The one-year risk of stent thrombosis and myocardial infarction was 1.8-fold and ~ 2.4-fold, respectively, higher in patients who developed CNR compared with patients who did not develop CNR. The increased risk of thrombotic events may be explained by blood stasis and prothrombotic milieu in the infarct-related artery territory in patients who develop CNR [21]. Although the risk for stent thrombosis did not reach the level of statistical significance, less patients with CNR received stents compared with patients with reflow. This procedural aspect may have reduced the occurrence of stent thrombosis among patients with CNR. On the other hand, the less frequent use of coronary stents in patients with CNR could have reduced the stabilization (pacification) effect of stents on atherosclerotic plaque [22] leading to more unstable treated lesions and increased risk of ischemic complications including myocardial infarction in the post-PCI period. In addition, patients with AMI who develop CNR have higher levels of several inflammatory markers in circulation, such as C-reactive protein [23], interleukin 6 (IL-6) [24] and interleukin 8 (IL-8) [25] compared with patients who did not develop CNR. Increased inflammatory burden is a well-known contributor to increased atherosclerotic plaque instability and higher risk of coronary events including myocardial infarction. The association between CNR and increased risk of bleeding remains poorly understood. However, patients with CNR were older (including the proportion of patients ≥ 75 years) than patients with reflow and a higher proportion of patients with CNR underwent PCI via femoral artery access known to be associated with a higher risk of bleeding compared with radial artery access [26]. Moreover, patients with CNR were more likely to receive aspirin loading before PCI, periprocedural glycoprotein 2b/3a inhibitors and oral anticoagulants at discharge compared with patients with reflow. Although, these factors could offer support for the finding of a higher risk of bleeding in patients with CNR in the setting of current study, whether CNR increases the risk for bleeding requires further investigation.

The observed difference in the trajectory of risk for adverse events associated with CNR is an interesting finding with potential clinical implications. Prior studies have assessed the short-term [9, 27, 28] and long-term [7, 8] risk for adverse events associated with CNR but they did not assess whether the risk associated with CNR differs over time. The landmark analysis showed that the risk for adverse events associated with CNR was highest within the first 30 days and almost plateaued between 30 days and 1 year after the index event. Conversely, the risk for adverse events increased steadily between 30 days and 1 year in patients with reflow. A recent study [29] showed a sharp decrease in survival within the first month and almost a parallel course of survival curves from 30 to 400 days after primary PCI in patients with STEMI providing support to our data. The early risk associated with CNR may be explained by at least two factors: the impact of CNR on myocardial hemodynamic and electrical stability and the duration of CNR after PCI. The development of CNR is associated with rapid hemodynamic and electrical instability, which is manifested clinically with ventricular arrhythmias [30], early congestive heart failure [6], cardiac rupture and early death from cardiac causes [31]. Expectedly, these events increase the early risk associated with CNR. Experimental and clinical studies have shown that CNR persists over days to weeks after the reperfusion. One study in rats showed that CNR persists up to one month after the reperfusion [32]. Schofer et al. [33] showed that scintigraphic zone of CNR persisted for 2–4 weeks after the intracoronary thrombolysis. Using myocardial contrast echocardiography, Galiuto et al. [34] showed that CNR observed within 24 h persisted at 1 month in 44% of patients with first AMI treated with thrombolysis or primary PCI. These studies clearly show that CNR and its deleterious effects on the myocardium persist over the 1st month after reperfusion in a sizeable proportion of patients with AMI.

This study has limitations. First, the study represents a retrospective, non-prespecified analysis and should be considered hypothesis generating. Second, CNR was investigator reported based on coronary angiography at the end of PCI procedure. Although CNR assessed in the catheterization laboratory may underestimate the frequency of CNR compared with core laboratory analysis, a recent study showed a higher risk for all-cause and cardiovascular mortality associated with investigator-based CNR than core laboratory-based CNR [29]. Investigator-based diagnosis of CNR in the catheterization laboratory may detect most severe and fixed microvascular obstruction over a large myocardial area/volume, which may explain the strong association with prognosis [29]. Third, the number of events for outcomes, such as stent thrombosis or stroke in patients with CNR was small. Thus, although the risk estimates suggested a higher risk associated with CNR for these outcomes, the level of statistical significance was not achieved. Fourth, the 1-year follow-up may not be long enough to assess clinical outcome after CNR. In particular, outcomes related to adverse left ventricular remodelling caused by CNR, may need more time to occur. Fifth, cardiac magnetic resonance, ST-segment resolution and myocardial blush data were not available for the analysis. Finally, the association between CNR and the primary outcome (NACCE) was adjusted for demographical and clinical variables; however, residual confounding cannot be ruled out.

In conclusion, in patients with AMI undergoing PCI with current DES and third generation P2Y_12_ receptor inhibitors, CNR was associated with a higher risk of adverse outcomes at 1 year. The risk associated with CNR appears to be greatest within the first 30 days after the reperfusion. These findings may have implications with respect to the risk stratification and care of patients with AMI who develop CNR, particularly in the first 30 days after the index event.

### Supplementary Information

Below is the link to the electronic supplementary material.

Supplementary file1 (PDF 338 KB)

## Data Availability

The data will be shared on reasonable request to the corresponding author.
