# Supplementary material for: Coronary no-reflow and adverse events in patients with acute myocardial infarction after percutaneous coronary intervention with current drug-eluting stents and third-generation P2Y12 inhibitors
Source: Clin Res Cardiol. 2023 Nov 14;113(7):1006–16. doi: 10.1007/s00392-023-02340-y (PMC11219448; doi:10.1007/s00392-023-02340-y)
Supplement: Supplementary file 1 — Supplementary file1 (PDF 338 KB) [file 392_2023_2340_MOESM1_ESM.pdf]

Supplementary Material

Coronary no-reflow and adverse events in patients with acute myocardial infarction after percutaneous coronary intervention with current drug-eluting stents and third-generation $P2Y_{12}$ inhibitors

Gjin Ndrepepa, MD[a], Salvatore Cassese, MD[a], Erion Xhepa, MD[a], Michael Joner, MD[a,b], Hendrik B. Sager, MD[a,b], Sebastian Kufner, MD[a], Karl-Ludwig Laugwitz, MD[b,c], Heribert Schunkert, MD[a,b], Adnan Kastrati, MD[a,b]

[a]Department of Cardiology, Deutsches Herzzentrum München, Technische Universität München, Munich, Germany
[b]German Center for Cardiovascular Research (DZHK), Partner Site Munich Heart Alliance, Munich, Germany
[c]Medizinische Klinik und Poliklinik Innere Medizin I (Kardiologie, Angiologie, Pneumologie), Klinikum rechts der Isar, Technische Universität München, Munich, Germany

Supplementary Table S1. Procedural characteristics

Characteristic	No-reflow (n=130)	Reflow (n=2970)	P value
Arterial access route			0.001
Femoral artery	92 (70.8)	1871 (63.0)	
Radial artery	34 (26.2)	1087 (36.6)	
Other	4 (3.0)	12 (0.4)	
Multivessel disease	87 (66.9)	1966 (66.2)	0.939
Angiographic LVEF (%)*	47.2 ± 11.4	50.9 ± 11.2	<0.001
Treated coronary artery			0.001
Left main	65 (2.2)	0 (0)	
Left anterior descending artery	1289 (43.4)	53 (40.8)	
Left circumflex artery	615 (20.7)	18 (13.8)	
Right coronary artery	958 (32.3)	51 (39.2)	
Bypass graft	43 (1.4)	8 (6.2)	
Complex lesions	90 (69.2)	1728 (58.2)	0.016
Preintervention TIMI flow grade			<0.001
0	92 (70.8)	1069 (36.0)	
1	15 (11.5)	258 (8.7)	
2	19 (14.6)	665 (22.4)	
3	4 (3.1)	978 (32.9)	
Postintervention TIMI flow grade			<0.001
0	30 (23.1)	0 (0)	
1	16 (12.3)	0 (0)	
2	84 (64.6)	0 (0)	
3	0 (0)	2970 (100.0)	
Type of PCI			
Drug-eluting stent	82 (63.1)	2720 (91.6)	<0.001
Bioresorbable vascular scaffold	3 (2.3)	173 (5.8)	
Plain balloon angioplasty	44 (33.8)	33 (1.1)	
Drug-eluting balloon	1 (0.8)	34 (1.2)	
Bare-metal stent	0 (0)	10 (0.3)	
Total stented length (mm)	32.3 ± 17.2	30.6 ± 16.9	0.364
Maximal stent diameter (mm)	3.13 ± 0.51	3.20 ± 0.50	0.228
More than 1 lesion treated	30 (23.1)	1044 (35.2)	0.006
Periprocedural drug therapy			
Aspirin loading	127 (97.7)	2685 (90.4)	0.008
Unfractionated heparin	125 (96.2)	2779 (93.6)	0.683
Bivalirudin	6 (4.6)	257 (8.6)	0.145
Low-molecular weight heparin	7 (5.4)	126 (4.2)	0.683
Glycoprotein 2b/3a inhibitors	44 (33.8)	372 (12.5)	<0.001

Data are counts (%) or mean ± standard deviation

LVEF=left ventricular ejection fraction; PCI = percutaneous coronary intervention; TIMI = Thrombolysis in Myocardial infarction

*Angiographic left ventricular ejection fraction was missing in 187 patients (3 patients with no reflow)

Supplementary Table S2. Drug therapy at discharge*

Drug	No-reflow (n=121)	Reflow (n=2920)	P value
Aspirin	113 (93.4)	2872 (98.4)	<0.001
Ticagrelor	52 (43.0)	1357 (46.5)	0.450
Prasugrel	47 (38.8)	1387 (47.5)	0.061
Clopidogrel	14 (11.6)	152 (5.2)	0.003
Oral antigoagulants	17 (14.0)	125 (4.3)	<0.001
Beta blocking agents	110 (90.1)	2545 (87.2)	0.224
Angiotensin-converting inhibitors	107 (88.4)	2545 (87.2)	0.681
Statins	115 (95.0)	2788 (95.5)	0.821

Data are counts (%)

*Therapy at discharge was not available in 9 patients with no-reflow and 50 patients with reflow.

Supplementary Table S3. Types of myocardial infarction according to 3rd Universal Definition of Myocardial Infarction

Type of myocardial infarction	No-reflow	
	Yes (n=11)	No (n=112)
Type 1 (n=70)	6 (54.5)	64 (56.9)
Type 2 (n=4)	0 (0)	4 (3.6)
Type 4a (n=20)	2 (18.2)	18 (16.1)
Type 4b (n=28)	3 (27.3)	25 (22.3)
Type 5 (n=1)	0 (0)	1 (0.8)

Data are counts (%). Overall P value for the differences between the groups was 0.944

Supplementary Table S4. Results of the multivariable Cox proportional hazards model applied to assess correlates of 30-day and one-year net adverse clinical and cerebral events

Variable	30-day NACCE		One-year NACCE	
	HR [95% CI]	P value	HR [95% CI]	P value
Coronary no-reflow	1.85 [1.17-2.93]	0.008	1.53 [1.01-2.33]	0.049
Drug (ticagrelor vs. prasugrel)	1.21 [0.93-1.56]	0.154	1.23 [1.01-1.51]	0.045
Age (for 1 year higher)	1.03 [1.02-1.04]	<0.001	1.03 [1.02-1.04]	<0.001
Women (vs. men)	1.94 [1.46-2.60]	<0.001	1.64 [1.30-2.08]	<0.001
Diabetes mellitus	1.06 [0.94-1.42]	0.670	1.24 [0.99-1.55]	0.061
Hypercholesterolemia	0.71 [0.54-0.93]	0.012	0.73 [0.59-0.91]	0.004
Systolic blood pressure (for 1 mmHg higher)	1.00 [0.99-1.01]	0.079	1.00 [1.00-1.01]	0.056
Heart rate (for 1 beat higher)	1.02 [1.01-1.03]	<0.001	1.01 [1.01-1.02]	<0.001
Serum creatinine (for µmol/l higher)	1.01 [1.00-1.02]	<0.001	1.01 [1.00-1.01]	<0.001
History of myocardial infarction	1.17 [0.85-1.66]	0.379	1.20 [0.91-1.58]	0.204
Multivessel disease	1.61 [1.15 -2.25]	0.005	1.53 [1.18-1.99]	0.001
Radial access (vs. femoral access)	0.56 [0.41-0.76]	<0.001	0.71 [0.57-0.90]	0.005
Target vessel (LAD artery)	1.19 [0.91-1.56]	0.196	1.12 [0.91-1.39]	0.289
LVEF (for 1% lower)	1.04 [1.02-1.05]	<0.001	1.03 [1.02-1.04]	<0.001
Complex lesions	1.05 [0.80-1.39]	0.717	1.17 [0.94-1.46]	0.169
Stenting (vs. balloon angioplasty)	0.58 [0.34-0.99]	0.045	0.75 [0.47-1.21]	0.253
Randomization to drug loading interval (for 1 minute shorter)	0.99 [0.99-1.00]	0.560	0.99 [0.99-1.00]	0.472

CI=confidence interval; HR=hazard ratio: LAD=left anterior descending; LVEF=left ventricular ejection fraction; NACCE= net adverse clinical and cerebral events

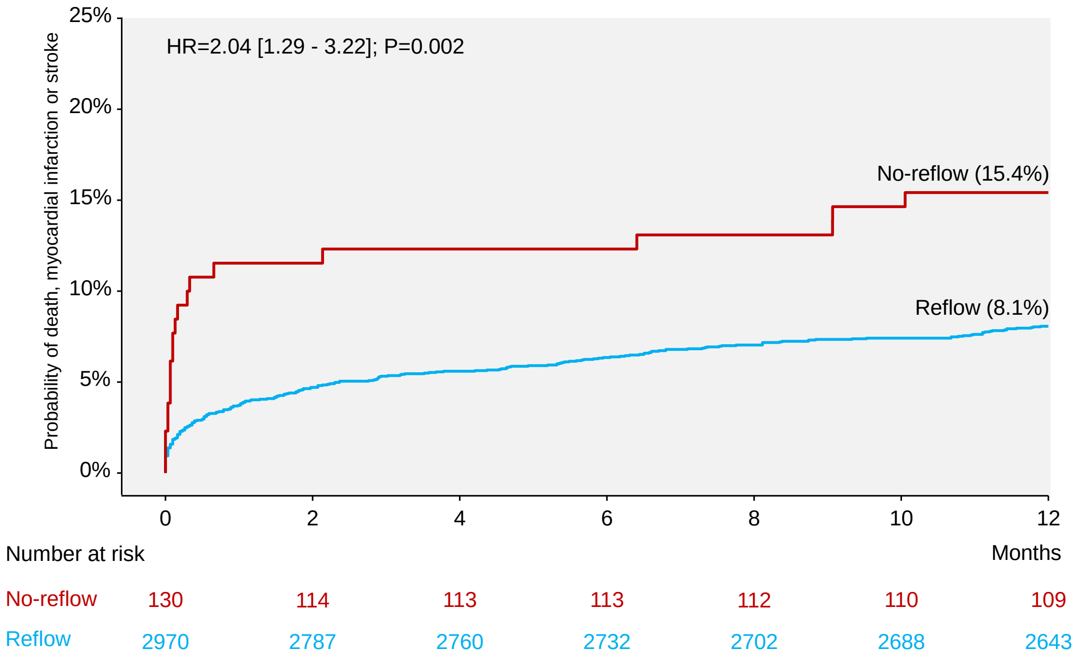

Supplementary Figure S1. One-year incidence of death, myocardial infarction or stroke in patients with coronary no-reflow and reflow. HR=hazard ratio.

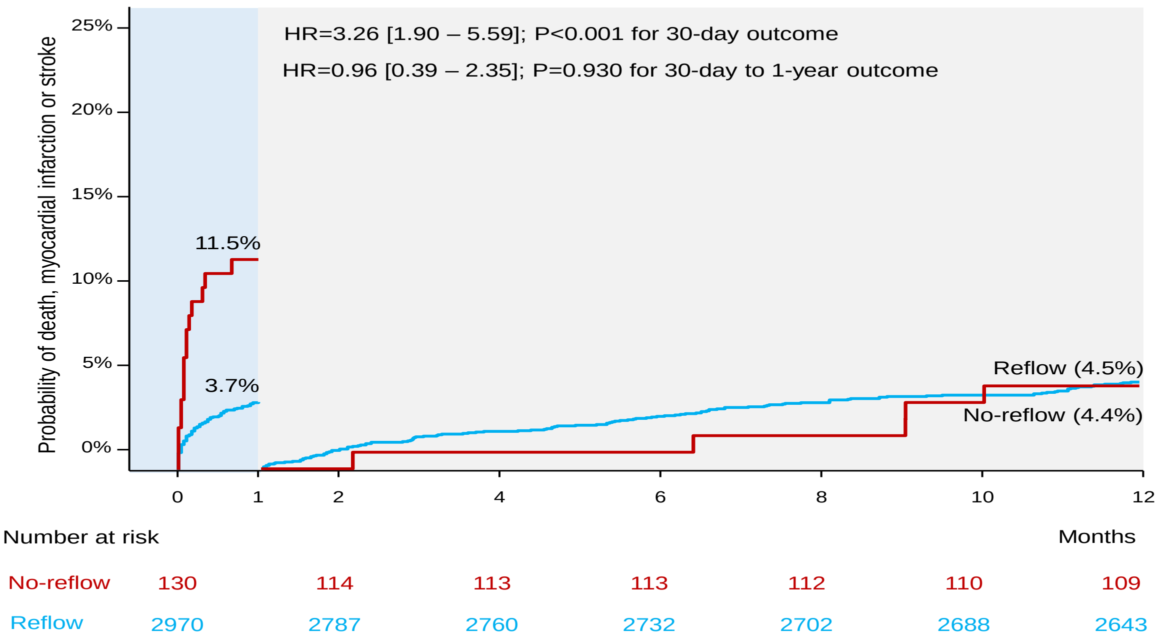

Supplementary Figure S2. Landmark analysis showing probability of early (within 30 days) and late (30 days to 1 year) occurrence of death, myocardial infarction or stroke in patients with coronary no-reflow and reflow. HR=hazard ratio.
